# Rotational restriction of nascent peptides as an essential element of co-translational protein folding: possible molecular players and structural consequences

**DOI:** 10.1186/s13062-017-0186-1

**Published:** 2017-05-31

**Authors:** Irina Sorokina, Arcady Mushegian

**Affiliations:** McLean, Virginia, USA

**Keywords:** Protein folding, Protein backbone twist, Protein backbone torsion, Peptide backbone twist, Peptide backbone torsion, Co-translational protein folding, Co-translational protein twist, Co-translational peptide twist, Trigger factor, Mechanism of chaperone action

## Abstract

**Background:**

A basic tenet of protein science is that all information about the spatial structure of proteins is present in their sequences. Nonetheless, many proteins fail to attain native structure upon experimental denaturation and refolding in vitro, raising the question of the specific role of cellular machinery in protein folding in vivo. Recently, we hypothesized that energy-dependent twisting of the protein backbone is an unappreciated essential factor guiding the protein folding process in vivo. Torque force may be applied by the ribosome co-translationally, and when accompanied by simultaneous restriction of the rotational mobility of the distal part of the growing chain, the resulting tension in the protein backbone would facilitate the formation of local secondary structure and direct the folding process.

**Results:**

Our model of the early stages of protein folding in vivo postulates that the free motion of both terminal regions of the protein during its synthesis and maturation is restricted. The long-known but unexplained phenomenon of statistical overrepresentation of protein termini on the surfaces of the protein structures may be an indication of the backbone twist-based folding mechanism; sustained maintenance of a twist requires that both ends of the protein chain are anchored in space, and if the ends are released only after the majority of folding is complete, they are much more likely to remain on the surface of the molecule. We identified the molecular components that are likely to play a role in the twisting of the nascent protein chain and in the anchoring of its N-terminus. The twist may be induced at the C-terminus of the nascent polypeptide by the peptidyltransferase center of the ribosome. Several ribosome-associated proteins, including the trigger factor in bacteria and the nascent polypeptide-associated complex in archaea and eukaryotes, may restrict the rotational mobility of the N-proximal regions of the peptides.

**Conclusions:**

Many experimental observations are consistent with the hypothesis of co-translational twisting of the protein backbone. Several molecular players in this hypothetical mechanism of protein folding can be suggested. In addition, the new view of protein folding in vivo opens the possibility of novel potential drug targets to combat human protein folding diseases.

**Reviewers:**

This article was reviewed by Lakshminarayan Iyer and István Simon.

**Electronic supplementary material:**

The online version of this article (doi:10.1186/s13062-017-0186-1) contains supplementary material, which is available to authorized users.

## Background

The protein folding problem has two main aspects. First, there is the question of the thermodynamic properties of the native protein structure. Second, there is a kinetic question about the set of steps, pathways and folding intermediates that proteins must go through to fold quickly and correctly. The fundamental assumption is that the complete set of instructions for correct protein folding is contained in the protein sequence, and therefore that studies of isolated protein molecules should provide most of the answers to these questions. The in vivo activities of intracellular molecular machines, such as ribosomes, chaperones, protein processing and quality-control systems, are thought to modulate these essential mechanisms, but the physical theory of those additional contributions is insufficiently developed and is not seen as key to understanding how proteins fold. As a result, several decades of theory, simulations, and laboratory work, based on the principle of sufficiency of sequence information for uncovering the protein folding pathways, have neither given us efficient algorithms for de novo folding of proteins, nor have provided much help to the practical efforts of protein purification and refolding.

**Fig. 1:**
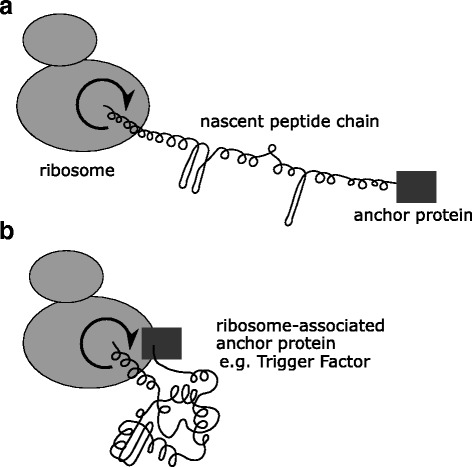
Schematic of protein backbone twist generated by the ribosome, sustained by the rotational restriction of the N-terminus, and inducing protein folding. A distal part of the protein must be anchored in order for the twist to be sustained and to facilitate the formation of secondary structure (**a**). As discussed in the text, anchoring proteins are associated with the ribosome itself in vivo (**b**)

Our recently proposed hypothesis of protein chain twist [1] posits that a torque force is applied to protein main chains co-translationally and post-translationally in vivo, and that such force is an important factor contributing to the speed and/or efficiency of protein folding to its native conformation*.* Generally speaking, if a point on a linear polymer molecule is restricted in mobility, then a twisting force applied to another point of the main chain will induce secondary structure in such a molecule (Fig. 1 and Additional File 1). Importantly, in order for a twist of the protein chain to be an essential component of the protein folding mechanism in vivo, the torque force should be applied to all proteins. One universal device that may be able to introduce a twist of all nascent peptide chains and thereby facilitate their subsequent folding is the ribosome itself [1].

In this communication, we specify the hypothesis by surveying the components of the ribosome and of the ribosome-associated machinery that are most likely to participate in generating a twist in the peptide chain. Examination of these details should improve our understanding of protein co-translational folding and of the physiological role of ribosome-associated chaperones. It also may help to explain the mystery of preferential surface exposure and statistical proximity of protein termini in three-dimensional protein structures, which may be seen as an inevitable side effect of the force and restraint applied on both ends of the protein chain throughout its synthesis and folding. We conclude with remarks about the aberrant early release of the protein chain termini as a possible cause of protein misfolding, and highlight this process as a possible drug target to combat protein folding diseases.

## Results and discussion

All plausible theories of protein folding in vivo require that a sufficient amount of secondary structure emerges early in the process of formation of the native protein fold. In our previous publication [1], we have noted that elements such as alpha-helices or beta-hairpins will be induced in a linear polymer if a torque force is applied to one point on its longitudinal axis while another, distal point on the same axis is restricted from rotating (Fig. 1 and Additional file 1). Here we discuss the evidence that the peptidyltransferase center of the ribosome constrains the C-terminus of the nascent peptide and may confer a twist to the nascent chain. In order for such a twist to persist in time and space, it is essential that a distal portion of the nascent peptide is itself topologically constrained, and we argue that application of such a constraint may be the common function of a cascade of factors associated with the ribosome, the nascent peptide and the freshly synthesized protein. The action of these factors enables co-translational folding, a phenomenon first recognized more than half a century ago, when the enzymatic activity of a heterodimeric enzyme was complemented in vitro by providing one component still attached to translating ribosomes [2], and now known to encompass a variety of molecular events.


Additional file 1: A toy model of a rotational twist applied to a string with spatially constrained positions of two distal parts, which induces complex and persistent topology. (MOV 15833 kb)


The hypothesis of co-translational twisting of the protein chain may shed new light on a long-standing puzzle of the peculiar behavior of the terminal regions in protein structures. In 1983, an estimate based on a limited sample suggested that the terminal residues of globular molecules tend to be physically closer to each other than might be expected [3]. A later analysis employing a more detailed random-expectation model and a larger set of structures suggested that there was no significant proximity boost of pairs of terminal residues, but the effect could be observed if six residues at each terminus were allowed to rearrange in space [4]. The trend persisted when a still larger collection of structures was examined and the alpha-carbon atoms in the most N-terminal and most C-terminal secondary structural elements were included [5]; the tendency appeared to be stronger for proteins that fold with two-state kinetics.

A confounding problem in those analyses is that protein termini may not be randomly positioned within the protein fold; if, for example, they tend to be solvent-exposed, their positions would be confined to a smaller set of spatial possibilities. This has been studied in detail with more than 400 proteins, and it was shown that the N-terminal and C-terminal residues, at least in small monomeric proteins, have a strong preference for being exposed on the surface of the molecule: the average solvent accessibility of terminal residues exceeds the accessibility of charged residues by 77% and accessibility of all residues by 142% [6]. Based on the half-maximal exposure, 80.3% of the N-terminal and 86.1% of the C-terminal residues are exposed, compared to 32% for all residues. Lattice-based simulations have shown that the tendency is stronger for models with a wider energy gap between the native and non-native conformations, and is strongest for the models that fold quickly in kinetic simulations (a proxy for the two-state folding mechanism) [6].

Various considerations have been put forward to explain these surface preferences of protein termini, several of them invoking co-translational protein folding [3–8]. In particular, Krishna and Englander [5] noted that

“*An apparent functional rationale for two-state folding, with the initial barrier being rate-limiting, is that it avoids the prolonged occupation of collapsed partially folded states that would expose proteins to unwanted intermolecular aggregation and proteolysis. This is desirable both during the initial folding process and subsequently during the life of the protein because native proteins repeatedly unfold and refold even under native conditions”,*

and, further,

*“To promote formation of a “correct” native topology initially and to avoid later fraying-dependent proteolysis and aggregation, it seems useful to correctly orient and tie down the chain ends in the initial folding-collapse step, keep them securely tied down in the native condition and in transient intermediates that form during folding and unfolding, and allow their release only in the final re-unfolding step*”.

The protein twist that we postulate requires precisely such a “tie-down” of protein ends. The implications of this, however, may be more profound than just a temporary, reversible decrease of the degrees of freedom proposed in ref. [5]. In fact, we believe that the entire protein co-translational folding process may occur while the ends of proteins are not free to move relative to one another, and preferential surface exposure of protein ends, as well as preferential N-to-C end proximity, may be inevitable mechanistic consequences of this process (for an animation modeling this phenomenon, see Additional file 1).

Although the question of co-translational protein folding has been intensely studied in recent years (reviewed in [8–11]), the anchoring of distal parts of the protein, and the possible role of such anchoring in protein chain twisting, has not been given much attention. We now discuss the features of the peptidyl transferase reaction and the properties of ribosome-associated factors that may play essential roles in these processes.

*Ribosome peptidyltransferase center (PTC)*. The large subunit of the ribosome performs a peptidyltransferase reaction, in which the peptidylated tRNA in the P-site undergoes aminolysis by the amino acid attached to tRNA in the A-site, and the carbonyl carbon of the peptide attaches to the amino group of that amino acid as a result. At the next steps, the deacylated tRNA from the P-site exits the ribosome through the E-site, the A-site tRNA is translocated to the P-site, and the nascent peptide is guided to the protein exit tunnel. Structural studies of ribosomal particles and of their complexes with substrate mimics [12–15] have revealed that as the peptidylated tRNA translocates from the A-site to P-site, it undergoes a nearly 180° rotation of its 3′-end [13]. This motion appears to be necessary for positioning the A-site nucleophilic amine and the P-site carbonyl carbon within the distance and into the topology that allow the transition state of the peptide bond to form, and may also be required for sending the nascent proteins into the exit tunnel [13–15]. It has been noted as a curiosity that the transition state geometry is achieved after a partial rotation of the tRNA 3′ end by only ≈45°, and that the role of the remaining swing is not clear [16].

The nascent peptide is thought to leave PTC and enter into the exit tunnel in an extended conformation [15–17]; this, however, appears to be at odds with the earlier theoretical stereochemical considerations, which concluded that the post-translocation bond geometry should be similar to what is observed in an alpha-helix [18].

*Ribosome exit tunnel*. The walls of the ribosome exit tunnel are made almost exclusively of ribosomal RNA. Parts of two proteins, however, protrude into the exit tunnel, and certain mutations in these proteins, L4 and L22, are associated with changes in resistance to macrolide antibiotics that bind to the tunnel interior [19, 20]. The better-studied L22 protein is thought to be part of a “gating factor” involved in macrolide interactions and in regulation of protein translation and sorting by sequence-dependent translation stalling; conformational rearrangements that involve the tip of a beta-hairpin within L22, potentially resulting in the tunnel occlusion, are thought to be involved in these effects [19, 21–23]. The roles of L4 in the tunnel, as well as of another protein, L39, located at tunnel opening, could be similar, though they are less well understood [19, 23, 24]. The observation that co-translational stalling in the presence of antibiotic may inhibit peptidyltrasferase reaction, depending on the identity of the tRNA in the A-site [25], also suggests a direct mechanistic connection between PTC and the constricted point of the exit tunnel. Taken together, the data above indicate that there is potential for protein chain twisting initiated at PTC but constrained at the distal end by various elements of the exit tunnel. This is compatible with the growing evidence that partial secondary structure is attained by various polypeptides inside the tunnel, and in some cases even of full tertiary structure adopted by small proteins in the tunnel or in the exit vestibule [26–28].

*Trigger factor.* Nearly all bacteria possess a general chaperone trigger factor (TF), consisting of an N-terminal ribosome-binding domain, a central peptidyl-prolyl isomerase (PPIase, or P) domain, and a C-terminal substrate-binding domain. The N-terminal and C-terminal domains fold together in space, forming an elongated body, and the P domain forms the head of the “dragon-shaped” overall structure [29]. The co-translational chaperone activity mainly resides in the body, where the two terminal domains form a cavity with a large internal surface suitable for interaction with the nascent peptides [30–32]. Structure-function studies have uncovered several activities of individual domains within TF. In particular, it has been shown that TF can:

dock onto the ribosomal L23 protein near the opening of the exit tunnel, interacting through a conserved “TF signature motif” [33];

restore viability of a synthetic lethal *tig-dnaK-* mutant, either fully when expressed as a whole protein, or partially when expressed as N + C or N + P domain fusions [34];

when on the ribosome, project the N- and C-terminal domains over the opening of the exit tunnel, presumably shielding nascent polypeptides from misfolding and degradation [30];

when off the ribosome and dimerized in the cytoplasm, bind to non-natively folded proteins, maintain them competent for refolding, and cooperate with other cytoplasmic chaperones to facilitate post-translational or post-stress protein folding [35];

presumably, expose hydrophobic patches in the cavity formed by the N- and C-domains to capture the hydrophobic segments of the nascent polypeptide emerging from the exit tunnel [36], though TF also appears to be recruited by proteins lacking linear hydrophobic segments and to bind to the folded form of a heavily charged ribosomal protein S7 [37].

Despite all this information, the fundamental question, i.e., “What is the mechanism by which TF affects the folding of its substrates in vivo?” was considered, by the experts in the field, to be still unanswered as recently as in 2010 (see in ref. [29]). An early indication that the mechanism might be complex was the demonstration that TF and DnaK, another general chaperone, work together to improve the folding yields of two newly translated multi-domain proteins, but delay the folding process both in vivo and in vitro while doing so [38]. On the other hand, TF has little effect on the co-translational folding of some small proteins, but does have an effect on the folding of a large single β-galactosidase domain, presumably through entangling the nascent chain with TF and slowing down its structural rearrangements [39]. Other studies have also observed that TF prolongs the lifetime of the extended, unfolded conformation of the bound nascent peptides, even suggesting that TF is an unfoldase rather than a folding agent [40].

The backbone twist hypothesis allows us to view these and other, seemingly conflicting, observations of TF’s mechanism of action in a new light. If protein folding to its native conformation requires that the secondary structure is induced at a local or global scale by application of torque force to the protein main chain, TF can be seen as one of the factors restricting the free rotational motion of the distal (in the first instance, N-terminal) regions of the peptide that is exiting the ribosome tunnel while a twist continues to be applied at to the C-terminus of the peptide in PTC. This restriction of the free rotation of the distal part of the molecule could be instrumental in inducing local secondary structure, and therefore in the overall folding process. This may be a general mechanism, applicable when either the unfolded or partially folded region of the protein is being anchored, and perhaps regardless of whether such a region is hydrophobic or charged (indeed, recent integration of structural data and molecular dynamics simulations suggest that TF is capable of binding to hydrophilic as well as hydrophobic regions [41]).

*Trigger factor homologs and analogs in archaea and eukaryotes?* Archaea and eukaryotes encode multiple PPIases, but are thought to lack sequence homologs of the N-terminal or C-terminal domains of bacterial trigger factors. Structural similarities, in the apparent absence of sequence-level homology, have been noticed between the C-terminal “cradle” domain of TF and a domain in bacterial periplasmic chaperone SurA [42]; interestingly, this region within SurA is fused to two PPIase domains, but they are not essential for SurA-mediated folding of an outer membrane protein [43]. Other bacterial proteins with similar structure have been characterized by sequencing and structural genomics efforts; several of them are also fused to PPIase domains and have chaperone-like activities [44].

We built probabilistic models of sequence conservation (profile-Hidden Markov Models; HMMs) for both terminal domains of bacterial TFs, and searched the database of the prebuilt HMMs models employing the HHPred server [45] as the search engine. When the HMM of the C-terminal domain of *Vibrio cholerae* trigger factor (PDB ID 1t11) was used as a query, and the entire set of HMMs built from each protein encoded by 13 complete archaeal genomes was used as the search space, the top-scoring match was to *Haloferax volcani* protein YP_003535684.1. The alignment extended to the complete length of the query, and the match had an E-value of 10^−9^, suggestive of a common evolutionary origin of this archaeal sequence and the bacterial TF C-domain (Additional file 2). Interestingly, YP_003535684.1 is annotated in the database as a PPIase and indeed contains a PPIase domain at the N-terminus; that domain did not contribute to the match we found, as the PPIase domain of the trigger factor was excluded from the query. BLAST searches of the sequence databases revealed the presence of clearly orthologous proteins in other archaea, mostly halophiles and methanogens. Thus, a fusion of PPIase and substrate-binding-like sequence domains appears to be not only widely distributed in bacteria, but is also found in a subset of archaea.

The structure of the N-terminal, ribosome-binding domain of TF also belongs to a fold shared by several other structural families, without an apparent unifying functional theme. The HHPred scan of the database of conserved domains detected a short site of specific sequence similarity between the N-terminal domain of TF and a region within signal recognition particle (SRP) subunit SecY/Sec61, represented in all domains of life. In TF, this similarity region corresponds to the ribosome-binding “signature motif” (Additional file 2). SRP, like TF, binds to ribosomal protein L23 [46], and the TF-like motif is likely to be the site of SRP interaction with the ribosome, just like its counterpart in TF. Similarity between limited regions of TF and this component of the signal recognition particle could be a case of molecular mimicry between two evolutionarily unrelated proteins; alternatively, this loop region may have been exchanged by DNA recombination between bacterial and archaeal ancestors.

Despite these newly found similarities, there are no orthologs of bacterial trigger factor in archaea and eukaryotes (other than bacterial-type trigger factors in plant chloroplasts). What, if anything, could play a universal TF-like role in the other two domains of life? One candidate is nascent polypeptide-associated complex (NAC), an evolutionarily conserved dimeric complex that had been first identified in yeast as a factor binding to nascent polypeptides as they emerge from ribosomes [47]. Eukaryotic NAC is a heterodimer of two non-identical subunits alpha and beta, which share a homologous sequence domain (NAC domain). In archaea, there is just one NAC-domain protein that is found as a homodimer in vivo, which, likewise, contacts the nascent chain on the ribosome. The fold of NAC subunits is unrelated to TF domains [48], but like TF and SRP, NAC binds to the ribosomal protein L23, in this case through a distinct RRK(X)nKK motif on the beta subunit [49].

Studies of NAC show parallels with the analysis of trigger factor function. Early on, NAC has been suggested to target proteins for membrane insertion, as it has been noticed that NAC detaches from the nascent peptide when a signal peptide fully emerges from the exit tunnel, and that NAC depletion from ribosomes results in mistranslocation of peptides into the endoplasmic reticulum [47]. Similarly, the first report of *E.coli* trigger factor also emphasized its role in converting a membrane protein into a membrane assembly-competent form [50]. As in the case of conditional synthetic lethal *tig-dnaK-* in *E.coli*, simultaneous deletion of genes encoding NAC and the Hsp70/40 system SSB-RAC in yeast causes conditional lethality [51]. These and other observations, such as broad sequence specificity of NAC binding to nascent chains [52], suggest a role for NAC as a general ribosome-associated chaperone. The mechanism of NAC action, and its possible role in anchoring the N-terminal region of a nascent protein while the C-terminus undergoes co-translational twisting, requires further investigation.

*Bacterial peptide deformylase and ubiquitous methionine aminopeptidases.* As indicated by the examples above, the ribosome may be viewed as a molecular platform, or perhaps a membrane-less intracellular compartment, responsible not only for protein synthesis, but also for spatially organizing many other important steps in protein folding, processing and sorting. The emerging N-terminal residue of bacterial proteins is typically formylmethionine, from which the formyl group is removed by peptide deformylase (PDF), and N-terminal methionine in all domains of life is removed by methionine aminopeptidases (MAPs). Both of these essential enzymes interact with nascent chains, at least in bacteria, and perform cleavage of the N-terminal groups at a chain length as short as 40 amino acids, suggesting that this happens while the enzymes are associated with the ribosome [53]. Moreover, PDF in *E.coli* can transiently bind to a crevice between proteins L22 and L32 [54]. In yeast, one MAP isoform appears to contact the ribosome at the universal adaptor site near L23, adjacent to the alpha subunit of NAC [55]. The crowdedness on the ribosome surface may afford additional opportunities to restrict the motions of the N-terminus or more internal portions of the nascent chain, at least temporarily, while its C-terminal portion is still being turned out by the PTC.

We surveyed the evidence in favor of the hypothesis that ribosome-associated chaperone systems serve in part to constrain the rotational mobility of the nascent peptide, while sustained rotational motion is applied to the C-terminus of the nascent peptide in PTC, thereby inducing a twist in the main chain that leads to formation of secondary structure. Some of the molecular components discussed above are found only in bacteria, others only in archaea and eukaryotes, and yet others are present universally, so that each biological species has more than one such system. Obviously, many other molecular machines are also coupled with translation and are involved in post-translational protein folding and sorting. We believe that these other, more “downstream” systems, such as the already-mentioned signal recognition particle, specialized secretion systems, and the networks of diverse classes of chaperones active in various cellular compartments, have their own connection to the protein twist hypothesis; this will be discussed elsewhere.

## Conclusions

Recently, single-molecule approaches have been used to study some of the forces that are applied to the nascent peptide during translation, for example pulling force (e.g., ref. [56]). Some of the forces involved in the interactions of the nascent peptide with chaperones have also been measured [57]. The existence of a torque component among the forces applied to the nascent peptide, however, has not yet been investigated. The framework of peptide chain anchoring and twisting, and the examples discussed in this paper, should suggest directions for experimental testing of these possibilities.

Co-translational protein folding that involves anchoring and twisting of the protein chain may be expected to require precise spatiotemporal coordination of peptide grip and release, and errors in this process may result in pathological states. In particular, this new view of protein folding in vivo could be instrumental for better understanding of the etiology of protein folding diseases and more generally proteostasis malfunction in eukaryotes. As one possibility, errors in co-translational protein twisting may result in a premature release of the terminal regions of proteins that are still undergoing folding, leading to accumulation of partially folded protein intermediates, which have been linked to proteostasis disorders [58–60]. If this is the case, then stabilization of interaction between the nascent protein and co-translational chaperones (recently dubbed ‘nascent chain welcoming committee’ [59]) may be tested as a strategy to reduce the accumulation of underfolded or misfolded protein intermediates in vivo. The hypothesis may be experimentally verified using existing models of folding diseases, most directly through chemical genetics, i.e., selection of the modified forms of cellular factors that impact protein release from the co-translational chaperone systems and identification of small molecules that affect these processes.

## Methods

Probabilistic searches of the prebuilt HMMs were performed using the HHPred server [45] as the search engine, with pdb70 + cdd, or all archaeal genomes selected as options for the search space. The searches were done in January of 2017. The identity of queries is discussed in the text and their list is given in Additional file 2.

## Reviewers’ comments

### Reviewer’s report 1: Lakshminarayan M. Iyer, National Center for Biotechnology Information, National Institutes of Health, Bethesda, USA

## Reviewer comments:

In this manuscript Sorokina and Mushegian build on their previous paper, which proposes an original idea, of the need for rotational restriction at the N-and C-termini of the nascent peptide during translation for efficient folding of proteins. Here they provide several possible candidates that could possibly aid the process. An important aspect of this publication is that the authors provide testable hypotheses by naming candidates that suit the role of anchors that provide rotational restrictions allowing for protein twisting and folding.

Author’s response: *We appreciate Dr. Iyer’s interest in our work, and his helpful comments and constructive criticism.*

## Reviewer comments, continued:

A few contrary points that come to mind that the authors might consider responding to. 1. A variety of proteins can be unfolded and refolded chemically (e.g. Ribonuclease A), suggesting that for many proteins, assisted folding is not necessary. Some protein purification methods in fact involve unfolding and refolding steps that is unaided. Since unassisted folding is the simplest form of protein folding, wouldn’t this be the likely ancestral state?

Author’s response: *Addressing first the problem of chemical unfolding and refolding and use of unassisted refolding in protein purification, we must emphasize that our knowledge of the ‘unfolded’ or ‘denatured’ state of proteins has been undergoing a profound change in recent years. This is worth a separate treatise, which is currently in preparation. We can summarize some of the relevant points here. First, it is becoming evident that for many proteins, what was for a long time considered their denatured state in fact includes persistent hydrophobic clusters and/or partial native-like secondary structure (see* [61] *for monographic treatment, in particular, Chapters 1 and 2). Second, biophysical studies of inclusion bodies, a common source of protein for purification by refolding, indicate that the proteins within these bodies are far from being denatured, and instead have highly ordered secondary structure and supramolecular organization* [62, 63]. *Protocols for protein refolding by denaturation-renaturation from such inclusion bodies are sensitive to the denaturation regime, often requiring intermediate concentrations of chaotropic agents* [64–66]. *This indicates that too high concentrations of chaotropic agents may hinder the success of refolding by destroying essential elements of secondary structure necessary for protein refolding.*

*We also agree that the ancestral state and evolution of the protein folding mechanism are worth pondering. If our hypothesis is correct, then the efficient folding of most, or all, proteins in the present-day cells requires a multicomponent, co-adapted molecular machinery, consisting not only of RNA but of many proteins that have to be well-folded themselves. The present-day protein sequences may be selected for efficient folding to their functional form after they have been “wound up” by this machinery of co-translational chain twisting. On the other hand, when such machinery in primitive life forms was not yet fully developed, protein sequences may have been selected for efficient adoption of their functional shape from a more random conformation, in the absence of the induced backbone twist. (All this, however, cannot be viewed separately from the mechanism of the ancient polypeptide synthesis, and any origins hypothesis that involves macromolecular interactions and geochemically plausible liquid-solid phase interfaces should consider rotational restriction during these processes).*

## Reviewer comments, continued:

2. Similarly, in proteins such as titin, individual domains (such as the Ig domain) can unfold and refold and do not seem to require any rotational restriction to achieve their folded state. Doesn’t this challenge the authors’ co-translational folding mechanism?

Author’s response: *To the contrary, refolding of the Ig domains in titin, which was studied by mechanical stretching and relaxation, using single-molecule techniques* [67–69], *produced a remarkable result: refolding in the tandemly repeated Ig-domain units was incomplete, and after repeated cycles of stretching-relaxation, ‘mechanical fatigue’ was observed, in which end-to-end length of the ‘refolded’ form continued to increase, without any change in the chemical environment* [70]. *In our opinion, this is evidence that repeated mechanical stretching destroys essential secondary or tertiary structure elements that were created by the cell machinery at the time of titin biosynthesis and maturation*.

*That said, single-molecule approaches, in particular magnetic tweezers, will undoubtedlybe useful indirect application and measurement of torque on the protein backbone. This should be one of the approaches to testing the hypotheses described in this work as well as in our earlier publication* [1].

## Reviewer comments, continued:

3. Enzymatic domains such as PPIases or Methionine aminopeptidases or peptide deformylase are predicted to function as N-terminal anchor domains that aid the predicted twisting process, by providing an N-terminal rotational restriction. However, enzymatic domains usually have a low associativity with their products. Shouldn’t the association/dissociation constants of these enzymes be considered to check if they show any unusual properties that supports the author’s hypothesis?

Author’s response: *Detailed understanding of reaction rates and binding constants is necessary for future kinetic modeling of co-translational protein folding. It is not clear to us whether the currently available values, typically obtained with single enzymes and model substrates in solution, are particularly illuminating; on the other hand, it is intriguing, for example, that E.coli peptide deformylase (Def) is inhibited by elevated concentrations of the substrate* [71], *implying higher associativity under these circumstances. In any case, the interaction of nascent peptides with trigger factor in bacteria is expected to occur on a longer time scale, in part due to kinetic trapping* [39], *as is presumably also the case for NAC interactions in archaea and eukaryotes. On the background of a slow, rate-controlling process, additional faster interactions could have modulatory or regulatory effects.*

## Reviewer comments, continued:

4. It would help their argument if the authors could analyze gene deletion data for the knockdown lethality of the genes of the proteins they discuss as potential candidates assisting protein folding.

Author’s response: *Great suggestion! In addition to the mention of tig-dnaK- synthetic lethality in E.coli in the main text, we are including more information from several other species in Additional file* *3* *.*

### Reviewer’s report 2: István Simon, Institute of Enzymology, Hungarian Academy of Sciences, Budapest, Hungary

## Reviewer comments

This is a follow up of the paper published by the same authors last year: Biol. Direct 2016; 11:64. It is a nice work, written in an easy to follow form. The possible effects of the rotational restriction of the nascent peptides in the course of protein biosynthesis are discussed for all the three kingdom of proteins. What I missed in the paper is a list of a few examples of forced twists in the X-ray structures and to see that proteins having these kind of structural elements are the same as the hard to refold proteins. I am also interested in the possible role of the signal peptides and the cases of zymogen protein in this folding phenomenon.

Author’s response: *We thank Professor Simon for his interest in our work and for his helpful comments. The question of forced twists in the secondary structures, as distinct from the regular turns in the alpha helices and* 3_10_ *helices, is interesting. There are hints suggesting that residual backbone twist is preserved even after the formation of the native tertiary structure; these include the known, but not well-understood, phenomenon of the twist observed in nearly every beta-sheet* [72], *as well as the fact that some of the loops in protein structures have been described as gently coiled, ever since this feature was noticed in lysozymes* [73, 74]. *More detailed analysis of these and similar structural features, as well as of their correlation with protein refolding efficiency, are planned for the near future*.

*The complexes that interact with signal peptides, i.e., signal recognition particles and translocon, are also of interest: much like the ribosome and its associated factors, each of these complexes can be both a protein-anchoring device and an energy source. SRP and SRP receptors are GTPases, and the translocon is an ATPase, but in all those cases, the energy balance of NTP hydrolysis is not well-studied (in the case of SRP, the role of GTP hydrolysis is not completely known, though it is thought to be required mostly for complex disassembly* [75]). *These complexes could be the additional components of the ‘protein welcoming committee’ enabling co-translational torsional restriction of the nascent protein chains, and/or providers of an extra energy boost for protein chain twisting*.

*Propeptide portions in many zymogens appear to play a role in folding of whole proteins or enzymatic domain chaperones* [76–78]. *Thus, propeptides are part of the chaperone repertoire of the cell, though they apparently are not general-purpose chaperones. The mechanisms discussed here, however, may have implications for the understanding of the function of this and several other classes of chaperones*.

## Additional files

Additional file 2: Selected HHPred results of sequence similarity searches with the N and C domains of trigger factor. (PDF 94 kb)

Additional file 3: Deletion phenotypes and genetic interactions of the ‘welcoming committee’ components in different species. (PDF 82 kb)
