# Supplementary material for: Rotational restriction of nascent peptides as an essential element of co-translational protein folding: possible molecular players and structural consequences
Source: Biol Direct. 2017 May 31;12:14. doi: 10.1186/s13062-017-0186-1 (PMC5452302; doi:10.1186/s13062-017-0186-1)
Supplement: Supplementary file 2 — Selected HHPred results of sequence similarity searches with the N and C domains of trigger factor. (PDF 94 kb) [file 13062_2017_186_MOESM2_ESM.pdf]

```
Q ss_pred                  HHHHHHHH-hCCCCCCCCCCCCHHHHHHHHCHH
Q 1t11 1-170:          33 AELRNIAK-NRRFDGFRKGKVPMKMVAKMYGKA   64 (170)
T COG0201             339 EIAENLKKSGGFIPGIRPGKDTEKYLNRVIPRL  371 (436)
T 5eul_Y              331   ADNLKKQGGYIPGIRPGKNTQEYVTRILYR  360 (424)
   T 3jc2_1           393     QLKEQQMVMRGHRETSM-VHELNRYI  417 (476)
   T ss_dssp                  HHHHHTCCCTTSCHHHH-HHHHHTTH
```

Combined results of HHPred search with the model of the ribosome-binding domain of trigger factor (TF) from Vibrio cholerae (PDB entry 1t11) suggests similarity between the ribosome-binding loop of TF and a segment in bacterial SecY subunit of signal recognition particle (COG0201, PDB 5eul_Y) as well as the eukaryotic homolog Sec61 (PDB 3jc2_1). Legend and color code are as in https://toolkit.tuebingen.mpg.de/hhpred/help_results (accessed on January 15, 2017) See text for the details.

```
Q ss_pred                 ccHHHHHHHHHHHHHHHHHHHHHHHHhCCCCCCHHHHHHHHHHHHHHHHHHHcCCCCCCcccccHHHHHHHHHHHHHHHHHH
Q 1t11 272-end:         2 KNMERELKSAIRNRVKSQAIEGLVKANDIDVPAALIDSEIDVLRRQAAQRFGGNEKQPLELPRELFEEQAKRRVVVGLLL   81 (162)
Q Consensus             2 ~~l~~~~~~~~~~~~~~~i~~~l~~~~~~~~iP~~~v~~~~~~~~~~~~~~~~~~~~g~~~~~~~~e~~~e~~~~~~a~~~vk~~lil   81 (162)
                          +++.............+.+++.+.+.+.++|++|+.++..+...+..+...+.+++..   +-.+..+++.|+++++..|++
T Consensus           198 ~~~~~~~~~~~~~~~~~~~~~~~~~e~~~ve~P~~lv~~e~~~~~~k~~~~~~~~~~~~~---~~~~~~~~~e~ae~rv~~~l~l  274 (306)
T YP_003535684.1    198 EQVVESDDDDEDAEPETETVTVEKDTLYIEATPQMTMNQQWMFSKQQIAQDIMQRL---DIDRVIVQETIEGGLGGMGGM  274 (306)
T ss_pred                  HHHHHHHHHHHHHHHHHHHHHHHHHhhCcccCCHHHHHHHHHHHHHHHHHHHHhhhcC---CccHHHHHHHHHhcccccHHH
Confidence                4567777888888888999999999999999999999888877665543332211 1   12255689999999999999
```

```
Q ss_pred                 HHHHHHcCCCCCHHHHHHHHHHHHHHHcCCh
Q 1t11 272-end:        82 GEVIRTNELKADEERVKGLIEEMASAYEDP  111 (162)
Q Consensus            82 ~~ia~~e~i~vt~eei~~~~~~~~~~~~~~~~~~~~~  111 (162)
                          .+|++.++|++|+++++..+..++..|..+
T Consensus           275 ~~i~~~~~~~ie~~~~e~v~~~~~~~~~~a~~~~~~~~  304 (306)
T YP_003535684.1    275 MGGAGGADIEEAIEDVDIDADELAAELDAD  304 (306)
T ss_pred                  HHHhhhccCcCCHHHHHHHHHHHHHHhcCCC
Confidence                999999999999999999999887643
```

Results of HHPred search with the model of nascent peptide-binding domain of trigger factor (TF) from Vibrio cholerae (PDB entry 1t11) suggests similarity with an archaeal peptidyl prolyl isomerase-containing protein that is a prototypic member of an archaeal protein family. Legend and color code are as in https://toolkit.tuebingen.mpg.de/hhpred/help_results (accessed on January 15, 2017). See text for the details.

Genbank IDs of archaeal peptidyl prolyl isomerases that contain domains homologous to the C-terminal peptide-binding domains of bacterial trigger factors.

490141180
399240172
1088762454
495658969
909649990
1097353159
1086400306
517069813
1103315236
494805044
970570542
811260002
521281767
1097623688
495849885
1094368135
445752961
506243483
445671409
495592617
909693565
495439252
541189638
1094712985
495368886
968521212
494484937
491112154
491118268
541203049
445645979
495599852
1090714506
490164541
910069402
968126923
909668242
491099079
1097658306
491708793
909674048
910073540
1103306079
505133720
445622068
541179776
910244091
493705138
1100834574
445621075
1086559941
397684285
910233034
909680708
493474967
968553123
961365054
910083988
492945767
1088842533
544639160
493699037
928944473
503806626
495701289
1054518949
1084275726
909660095
490730012
491677802

557612670
490156157
499542727
494348647
499642524
909709337
910009483
493185377
972339273
490388342
495584155
490650462
851124370
910015590
495016547
492981202
503646618
495861375
496124926
491746797
1086502794
909681785
910245949
909686649
1054611524
506268508
927059285
495274075
493053887
1094765193
1097600219
490151466
495721178
909667367
506389838
493678473
909645146
910055063
909664823
499205397
493881288
493048131
1097140905
910226582
494168621
910217224
1088885398
493714246
495693086
1086674587
541183957
1120447185
1093238166
541192759
494966902
493012154
1097332625
543416520
519066373
494970764
433290203
515912421
505221295
495269253
490735872
910076220
494587674
493940013
1054648360
495279432
495800910
492957479
557609599
494468144
557617505

495640872
1086453537
495258116
1086337368
1093226184
502707654
496170956
491180236
1097797657
1054060765
972326436
494239175
557615457
1086429121
493723439
972470398
541197574
557372265
504369155
499891080
541186355
910052715
497447154
503818899
541203803
937364833
928923306
1094265107
851153762
938511716
91711388
850996725
1001923315
851355850
1119383801
502802718
1119641404
1094219534
926635982
567125526
757130585
973024689
729326913
1003712717
1008951143
1008951143
729315994
87294884
1070294278
115396981
523576919
523576919
523576919
780676884
