# Supplementary material for: Rotational restriction of nascent peptides as an essential element of co-translational protein folding: possible molecular players and structural consequences
Source: Biol Direct. 2017 May 31;12:14. doi: 10.1186/s13062-017-0186-1 (PMC5452302; doi:10.1186/s13062-017-0186-1)
Supplement: Supplementary file 3 — Deletion phenotypes and genetic interactions of the ‘welcoming committee’ components in different species. (PDF 82 kb) [file 13062_2017_186_MOESM3_ESM.pdf]

Select information on the essentiality of the 'welcoming committee' members in different species

	Trigger factor	Peptide deformylase	Methionine aminopeptidase	NAC alpha	NAC beta
**Bacteria**					
Acinetobacter baumannii ATCC 17978		DEG10430036	DEG10440101	n/a	
Acinetobacter baylyi ADP1		DEG10130085<br>DEG10130031		n/a	
Bacillus subtilis 168			DEG10010057	n/a	
Bacillus thuringiensis BMB171	DEG10470404 *			n/a	
Bacteroides fragilis 638R			DEG10340460	n/a	
Brevundimonas subvibrioides ATCC 15264		DEG10460100	DEG10460393	n/a	
Campylobacter jejuni subsp. *jejuni* NCTC 11168		DEG10310020		n/a	
Caulobacter crescentus		DEG10200038	DEG10200355	n/a	
Escherichia coli K-12	Synthetic lethal with dnaK at the elevated temperatures (see text)			n/a	
Escherichia coli MG1655 I		DEG10180208<br>DEG10180488	DEG10180038	n/a	
Escherichia coli MG1655 II		DEG10190202	DEG10190027	n/a	
Escherichia coli ST131 strain EC958			DEG10480032	n/a	
Francisella novicida U112		DEG10120018	DEG10120124	n/a	
Haemophilus influenzae Rd KW20		DEG10050569	DEG10050631	n/a	
Helicobacter pylori 26695		DEG10080137	DEG10080251	n/a	
Mycobacterium tuberculosis H37Rv		DEG10100057		n/a	
Mycobacterium tuberculosis H37Rv II		DEG10250082	DEG10250554	n/a	
Mycobacterium tuberculosis H37Rv III			DEG10270514	n/a	
Mycoplasma genitalium G37		DEG10060087	DEG10060143	n/a	
Mycoplasma pulmonis UAB CTIP		DEG10140270	DEG10140220	n/a	
Alphaproteobacterium NC_003062		DEG10450033		n/a	
Porphyromonas gingivalis ATCC 33277			DEG10220400	n/a	
Pseudomonas aeruginosa PAO1	DEG10300030	DEG10360008	DEG10300061	n/a	
Pseudomonas aeruginosa UCBPP-PA14		DEG10150005		n/a	
Rhodopseudomonas palustris CGA009		DEG10410136	DEG10410100	n/a	
Salmonella enterica serovar Typhi		DEG10160328	DEG10160032	n/a	
Salmonella enterica serovar Typhi Ty2		DEG10330332	DEG10330033	n/a	
Salmonella enterica serovar Typhimurium SL1344		DEG10320262	DEG10320032	n/a	
Shewanella oneidensis MR-1		DEG10290147	DEG10290149	n/a	
Sphingomonas wittichii RW1		DEG10280133;<br>DEG10280464		n/a	
Staphylococcus aureus N315			DEG10020214	n/a	
Staphylococcus aureus NCTC 8325		DEG10170112	DEG10170270	n/a	
Streptococcus agalactiae A909		DEG10420285	DEG10420148	n/a	
Streptococcus pneumoniae		DEG10070178	DEG10070164	n/a	
Streptococcus pyogenes MGAS5448		DEG10370208	DEG10370144	n/a	
Streptococcus pyogenes NZ131			DEG10380158	n/a	
Streptococcus sanguinis		DEG10210191	DEG10210132	n/a	
Synechococcus elongatus PCC 7942	DEG10400655	DEG10400332	DEG10400635	n/a	
Vibrio cholerae N16961			DEG10030451	n/a	
**Archaea**					
Methanococcus maripaludis S2	n/a	n/a	DEG30010413		
**Eukaryotes**					
Saccharomyces cerevisiae	n/a	n/a	Two paralogs, YLR244C and YBL091C; each single deletion grows slowly, double deletion is lethal **	YHR193C, not essential***	Two paralogs YPL037C and YDR252W, form synthetic lethal at the elevated temperature***
Homo sapiens	n/a	n/a	DEG20120885<br>DEG20130932<br>DEG20140805<br>DEG20170620<br>DEG20190808<br>DEG20201013<br>DEG20230745<br>DEG20240957 (METAP1)<br>DEG20100715<br>DEG20111958<br>DEG20120886<br>DEG20130933<br>DEG20170621<br>DEG20190809<br>DEG20201014<br>DEG20210910<br>DEG20230746<br>DEG20240958 (METAP2)	DEG20102305<br>DEG20121007<br>DEG20131051<br>DEG20220167	

* DEG identifiers are from the Database of Essential genes (Luo H, Lin Y, Gao F, Zhang CT and Zhang R. DEG 10, an update of the Database of Essential Genes that includes both protein-coding genes and non-coding genomic elements. Nucleic Acids Research 2014:42; D574-D580. http://www.essentialgene.org/). Multiple DEG numbers in one cell indicate independent screening experiments.

** Li X, Chang YH. Amino-terminal protein processing in *Saccharomyces cerevisiae* is an essential function that requires two distinct methionine aminopeptidases. Proc Natl Acad Sci U S A. 1995;92:12357-12361.

*** Reimann B, Bradsher J, Franke J, Hartmann E, Wiedmann M, Prehn S, Wiedmann B. Initial characterization of the nascent polypeptide-associated complex in yeast. Yeast. 1999;15:397-407.
